# The feasibility of targeting macrophage for disease treatment: roles of CEBPD

**DOI:** 10.3389/fimmu.2025.1650161

**Published:** 2025-09-08

**Authors:** Tian Fan, Shaoling Lin, Jingjing Zhou, Jia Chen, Lexun Wang

**Affiliations:** ^1^ School of Life Sciences, Guangzhou University, Guangzhou, China; ^2^ Guangdong Metabolic Diseases Research Center of Integrated Chinese and Western Medicine, Guangzhou, Guangdong, China; ^3^ Key Laboratory of Glucolipid Metabolic Disorder, Ministry of Education of China, Guangzhou, Guangdong, China; ^4^ Guangdong Key Laboratory of Metabolic Disease Prevention and Treatment of Traditional Chinese Medicine, Guangzhou, Guangdong, China; ^5^ Institute of Chinese Medicine, Guangdong Pharmaceutical University, Guangzhou, Guangdong, China; ^6^ Beijing Key Laboratory of Maternal-Fetal Medicine and Fetal Heart Disease & Echocardiography Department, Beijing Anzhen Hospital, Capital Medical University, Beijing, China; ^7^ School of Chinese Medicine, Guangdong Pharmaceutical University, Guangzhou, China

**Keywords:** macrophage, CEBPD, polarization, phagocytosis, atherosclerosis, osteoporosis

## Abstract

As ubiquitous innate immune cells, macrophages are crucial for tissue homeostasis and disease pathogenesis. Although our understanding of macrophage subsets and functions has advanced, no effective strategies are available for targeting macrophages to treat diseases in clinical settings due to their heterogeneity. Transcription factors that regulate macrophage function have received increasing attention. CCAAT/enhancer-binding protein delta (CEBPD), an inflammation-associated transcription factor characterized by low basal expression but rapid induction by stimuli, has emerged as a key regulator of macrophages. CEBPD governs diverse biological processes in macrophages through its target genes. Furthermore, macrophage CEBPD significantly contributes to various pathologies. Modulating CEBPD expression or activity in macrophages could regulate various molecular processes to improve disease progression and alleviate organ damage; therefore, novel CEBPD-based therapeutic methods for treating diseases have attracted attention. In this review, we describe the factors upstream and downstream of CEBPD in macrophages. We then summarize recent advances in the regulation of macrophage biological processes by CEBPD. Finally, we discuss the contribution of macrophage CEBPD to various diseases and highlight strategies for developing novel therapies to modulate macrophage function by targeting CEBPD.

## 1 Introduction

Macrophages are crucial innate immune cells present in essentially all tissues and play vital roles in tissue development, homeostasis, and pathogenesis (1). In the past decade, macrophages have been established as two broad populations originating from either embryonic or definitive bone marrow-derived hematopoiesis, and macrophages in most tissues consist of these two populations (2, 3). Embryo-derived macrophages, termed tissue-resident macrophages (TRMs), are characterized by their persistence in adults and their stable and close association with tissue cells (3). Monocyte-derived macrophages (MDMs) originating from bone marrow hematopoietic stem cells are short-lived, rely on circulating monocytes for renewal, and can expand significantly in response to multiple stimuli (4). In certain tissues such as the brain, epidermis, and liver, TRMs maintain themselves by self-renewal, whereas TRMs in the gut, dermis, lung, and spleen are replaced by monocytes at tissue-specific levels (5, 6). Functionally, TRMs integrate signals from environmental sensors to orchestrate adaptive cellular responses critical for the growth and homeostasis of tissue cells, whereas MDMs play an important role in pathological conditions including inflammation, fibrosis, infection, and malignant remodeling (3, 4, 6). Recent advances in single-cell RNA sequencing have revealed that these two types of macrophages have different transcriptomes (7–9), which may underlie their different functions.

Although our knowledge of macrophage subsets and functions has advanced significantly, no effective strategies are available for targeting macrophages to treat diseases in clinical settings. Existing strategies mainly target neutralization of factors secreted by macrophages, including interleukin-1 beta, but this is not directly related to targeting macrophages (8, 9). This may be attributed to the following reasons: (1) Macrophage plasticity changes according to the surroundings, making the targeting of a particular subset of macrophages for disease treatment much less feasible. Increasing data show that macrophage types and compositions change considerably at different times or stages of the disease process (7, 10, 11). (2) The identification of macrophage phenotypes relies on multiple rather than single markers (12, 13). Further, the relationship between these markers and macrophage functions is unclear and requires further study. (3) Technically, protein engineering techniques and targeted delivery systems have a long way to go before diseases can be treated by targeting macrophages. These techniques play an increasingly important role in tumor therapy; however, they mostly induce immune cells to kill tumor cells rather than target the immune cells themselves (14, 15). In mice and humans, genetically inherited macrophage defects often result in severe or even fatal disorders, indicating that targeting macrophages should involve modulating, not just inhibiting their function (3, 16, 17).

**Figure 1:**
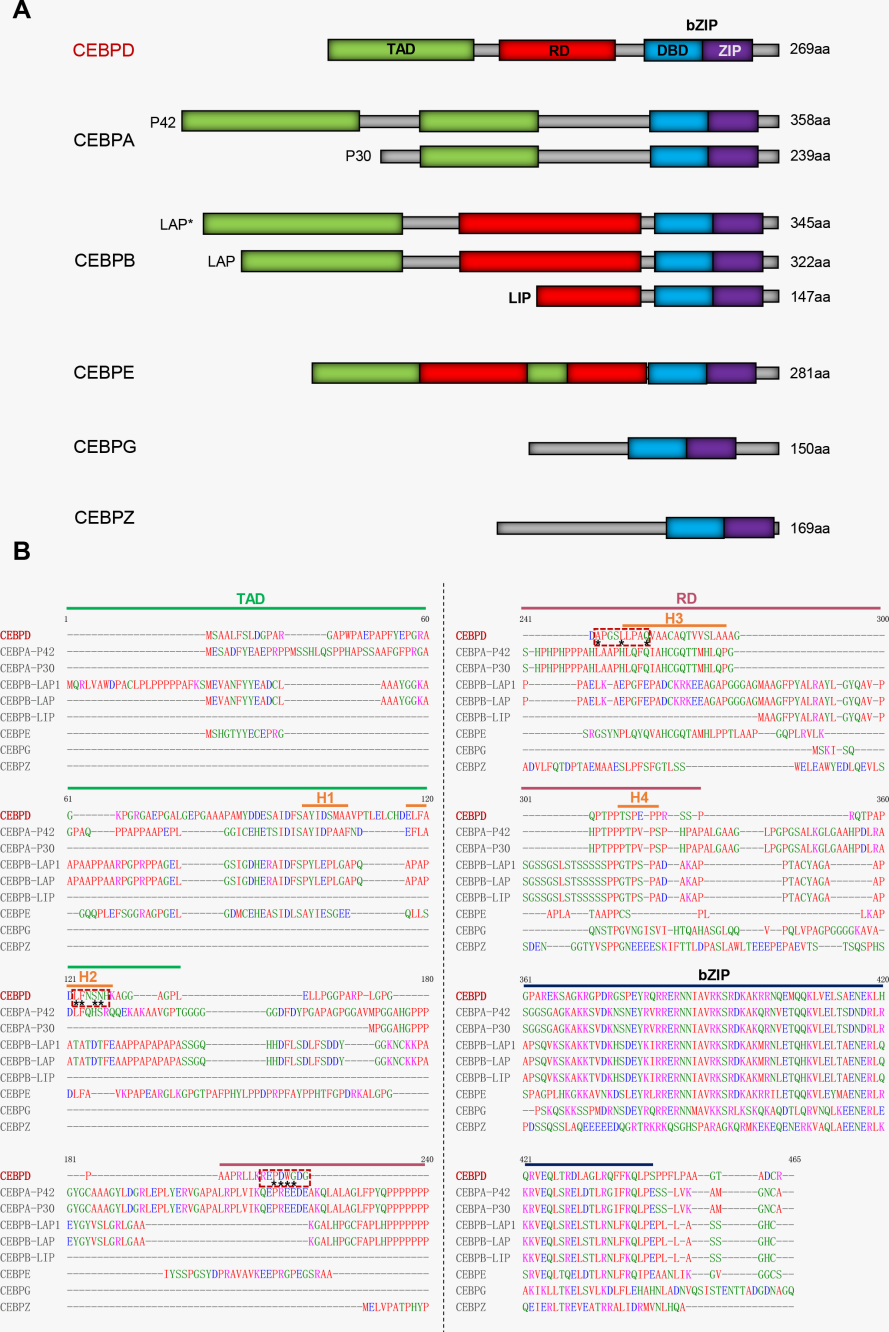
Comparative analysis of protein sequence between CEBPD and other CEBP family members. **(A)** The cartoon of domains among CEBP family members. **(B)** The red dashed box indicates the main region on CEBPD protein where the compound binds to. Asterisks indicate main points of contact of CEBPD. H, helix; TAD, Transcriptional activation domain; RD, Regulatory domain; bZIP, Basic-leucine zipper domain; DBD, DNA-binding domain; ZIP, Leucine zipper.

CCAAT/enhancer-binding protein delta (CEBPD) belongs to the CEBP family of the basic-leucine zipper (bZIP) class of transcription factors. Six members of this family share a highly conserved C-terminal region comprising a basic amino acid-rich DNA-binding motif, followed by a bZIP domain responsible for dimerization (**Figure 1A**) (18). Under normal physiological conditions, CEBPD expression is low in various cells including macrophages, but it can be rapidly induced by external stimuli (19).

Many studies have shown that CEBPD plays a crucial role in pathological processes including tumorigenesis and inflammatory responses, as well as neurological and fibrotic diseases, and these effects have been excellently reviewed elsewhere (19–22). CEBPD is involved in these pathological processes mainly by regulating its target genes. Recent studies have shown that macrophage CEBPD participates in the progression of various diseases including osteoporosis, rheumatoid arthritis (RA), atherosclerosis, lung injury, and cancers (23–27). Overexpression of CEBPD in macrophages, caused by mitochondrial dysfunction, directly upregulates the expression of cathepsin K (CTSK), which induces macrophage differentiation into osteoclasts, leading to bone loss (28). In RA, CEBPD is induced and upregulated in macrophages, enhancing the tube formation of endothelial cells and the migration and proliferation of synoviocytes to promote RA progression (24, 29). Moreover, activated macrophage CEBPD promotes tumor progression and atherosclerosis (25, 27). In response to external stimuli, upregulated CEBPD in macrophages can modify various cellular biological processes, including inflammatory response, oxidative stress, phagocytosis, and pyrimidine metabolism (25, 30–32). Furthermore, CEBPD plays a crucial role in macrophage polarization (30, 33). These studies indicate that macrophage CEBPD may be a potential target for disease treatment.

In this review, we first discuss the factors upstream and downstream of CEBPD in macrophages and the regulation of macrophage biological processes by CEBPD. We then describe the role of macrophage CEBPD in various diseases. We also discuss the feasibility of targeting macrophage CEBPD for disease treatment.

## 2 The upstream and downstream factors of CEBPD in macrophages

As a transcription factor, the main role of CEBPD is to regulate the expression of downstream target genes involved in various cellular biological processes. The transcriptional function of CEBPD also depends on the regulation of its expression and activity by upstream factors. In this section, we describe the upstream and downstream factors of CEBPD in macrophages.

### 2.1 Upstream regulators of CEBPD

Previous studies indicate that CEBPD expression in macrophages is regulated at three levels: gene transcription, mRNA stability, and protein expression. Recent studies have shown that some transcription factors, such as activator protein 1 (AP-1), cAMP-response element binding protein (CREB), and specificity protein-1 (SP-1), can bind to the CEBPD gene promoter and directly upregulate its expression in macrophages (25, 34, 35). Further, CEBPD regulates its expression in a positive feedback manner by binding to its own promoter (36). In contrast, activating transcription factor 3 (ATF3) directly binds to the CEBPD gene promoter and represses its expression in macrophages (36). Regarding CEBPD mRNA stability, the RNA-binding protein Hu antigen R (HuR) can bind to the 3’-untranslated region (3’-UTR) of CEBPD mRNA and stabilize it, thereby increasing its expression in macrophages (37). MicroRNAs (miRNAs) form a class of small noncoding RNAs that regulate gene expression through degradation or inhibition of specific mRNA targets by binding to their 3’-UTR (18). MiRNA let-7c directly inhibits CEBPD expression by promoting its mRNA degradation (30). At the protein level, ubiquitin-ligase F-box and WD repeat domain containing 7 α (FBXW7α) can bind to CEBPD to promote its degradation through ubiquitylation in macrophages (38). In addition, phosphatidylinositol-3-kinase (PI3K) and mitogen-activated protein kinases (MAPK) promote the nuclear translocation of CEBPD to activate its transcriptional function (39). The upstream regulators of CEBPD in macrophages are presented in detail in **Table 1**.

**Table 1:** Upstream regulators of CEBPD in macrophages.

Regulators	Effects (+: positive; -: negative)	References	
Gene transcription			
AP-1	Upregulates the transcription of CEBPD gene (+)	(34)	
ATF3	Inhibits the transcription of CEBPD gene (-)	(36)	
CEBPD	Upregulates its gene transcription (+)	(36)	
c-JUN	Upregulates the transcription of CEBPD gene (+)	(40, 41)	
CREB	Upregulates the transcription of CEBPD gene (+)	(25)	
c-REL	Upregulates the transcription of CEBPD gene (+)	(40, 41)	
EGR-1	Upregulates the transcription of CEBPD gene (+)	(42)	
SP-1	Upregulates the transcription of CEBPD gene (+)	(35, 40)	
mRNA stability			
HuR	Binds to and stabilizes CEBPD mRNA to promote CEBPD expression (+)	(27, 37)	
Let-7c	Binds to the 3’-UTR of CEBPD mRNA to promote its degradation (-)	(30, 33)	
Let-7-5p	Binds to the 3’-UTR of CEBPD mRNA to promote its degradation (-)	(43)	
Protein stability and activity			
FBXW7α	Binds to CEBPD to promote degradation through ubiquitylation (-)	(38)	
GSK-3β	Phosphorylates CEBPD at T156 for its degradation (-)	(38)	
PI3K	Promotes nuclear translocation of CEBPD (+)	(39)	
MAPK	Promotes nuclear translocation of CEBPD (+)	(39, 44)	

### 2.2 Downstream targets of CEBPD

To date, 35 target genes of CEBPD have been identified in macrophages (**Table 2**). Based on their function, these genes can be divided into 9 categories: inflammatory and immune response, material transport, oxidative stress, cell pyroptosis, differentiation, blood coagulation, coagulation and complement system, extracellular matrix, and ubiquitination. Of these, 60% (21/35) are associated with inflammatory and immune responses (**Table 2**). CEBPD binds to the promoter of most target genes and upregulates their expression. However, CEBPD binding to the promoter of ATP-binding cassette subfamily A member 1 (ABCA1), major histocompatibility complex class II trans-activator (CIITA), and FBXW7α inhibits expression of these genes in macrophages (25, 38, 48). The inhibitory effects of CEBPD may be due to its formation of heterodimers with negative regulatory factors such as its CEBP family member CCAAT/enhancer-binding protein gamma (CEBPG) (49). In addition to the well-defined target genes in macrophages, many additional targets of CEBPD have been reported in other cells (18). In fact, the number of CEBPD target genes is extensive, as shown in target gene online analysis databases (such as Transcription Factor Target Gene Database, Gene Transcription Regulation Database, Harmonizonme, and Transcriptional Regulatory Relationships Unraveled by Sentence-based Text mining). Further investigations will be required to determine whether these genes are regulated by CEBPD in macrophages.

**Table 2:** Downstream target genes of CEBPD in macrophages.

Gene	Positive (+) or negative (-) regulation	References	
Inflammatory and immune response			
ALOX5AP	(+)	(45)
CAMP	(+)	(36)
CCL20	(+)	(24)
CCL3	(+)	(36)
CD274	(+)	(42)
CEBPD	(+)	(36)
COX2	(+)	(34, 39, 46, 47)
CIITA	**(-)**	(48)
CXCL1	(+)	(24)	
CXCL2	(+)	(36)	
IL6	(+)	(36, 49)	
IL10	(+)	(27)	
IL23A	(+)	(24)	
PTX3	(+)	(25, 27, 37, 50)	
S100A8	(+)	(51)	
S100A9	(+)	(51)	
SAA3	(+)	(36)	
TLR4	(+)	(38)	
TLR8	(+)	(52)	
TLR9	(+)	(53)	
TNFα	(+)	(49, 54)	
Material transport			
ABCA1	**(-)**	(25)	
CP	(+)	(36)	
HP	(+)	(36)	
Oxidative stress			
NOS2	(+)	(47)	
NOX1	(+)	(32)	
Cell pyroptosis			
GSDMD	(+)	(55)	
GSDME	(+)	(55)	
Cell differentiation			
CSF1R	(+)	(35)	
CTSK	(+)	(28)	
Coagulation and complement system			
C3	(+)	(36)	
F10	(+)	(36)	
SERPINB2	(+)	(36)	
Others			
FBXW7α	**(-)**	(38)	
TNFAIP6	(+)	(24, 36)	

## 3 Regulatory roles of CEBPD in macrophage biological processes

**Figure 2:**
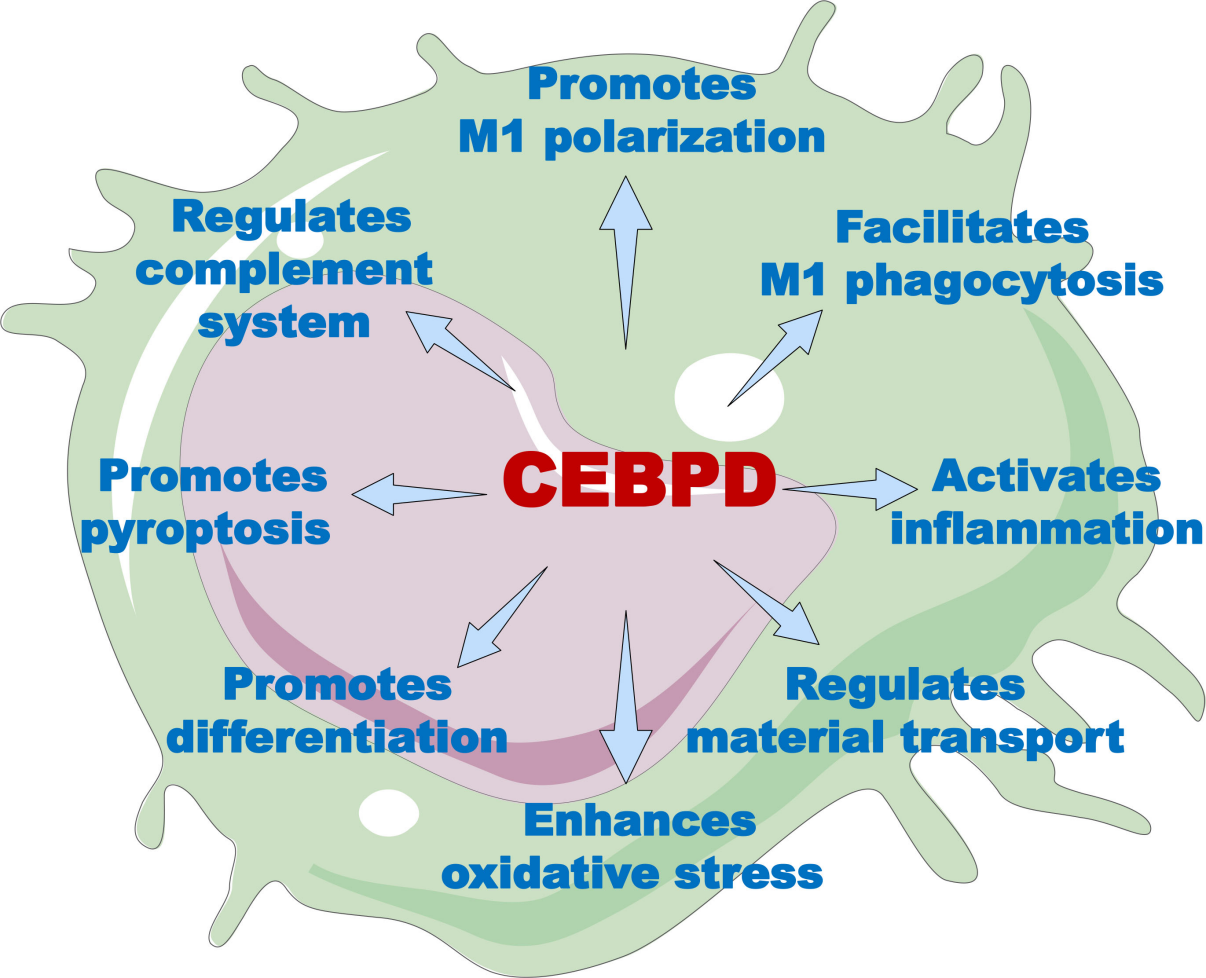
The overview of CEBPD functions in macrophages. Through its target genes, CEBPD can modulate a variety of macrophage functions, including polarization, phagocytosis, inflammatory response, substance transport, oxidative stress, cell differentiation, cellular pyroptosis, and complement activity.

Macrophages are among the first responders of the immune system. In this role, they have both effector functions for neutralizing pathogens and sentinel functions for alerting other immune cells to diverse pathological threats, thereby initiating and coordinating a multipronged immune response through the secretion of factors (56). CEBPD participates in regulating the multiple biological processes in macrophages under external stimuli (**Figure 2**, **Supplementary Table S1**). In this section, we discuss the roles of CEBPD in macrophage polarization, phagocytosis, inflammatory responses, oxidative stress, cell pyroptosis, and differentiation.

### 3.1 Macrophage polarization

Macrophage polarization is a process in which macrophages acquire distinct effector states based on surrounding microenvironmental stimuli to perform multiple and sometimes opposite functions. Traditionally, macrophages were thought to be polarized into two opposite types: classically activated or pro-inflammatory macrophages (M1) and alternatively activated or anti-inflammatory macrophages (M2) (12). However, recent advances have revealed a spectrum of macrophage activation states *in vivo* that extend beyond this simple dichotomy (57). Nevertheless, this dichotomy still reflects, to some extent, the relationship between different polarization states (M1 or M2) and the disease process.

Various studies have shown that CEBPD mRNA and protein levels are increased in macrophages following lipopolysaccharide (LPS) treatment *in vitro*, suggesting that CEBPD participates in macrophage M1 polarization (46, 58). Furthermore, CEBPD upregulation caused by circular RNA circCdyl (M1-enriched) overexpression is positively correlated with M1 polarization (30). On the other hand, inhibition of CEBPD by let-7c or interfering RNA induces macrophage polarization towards M2 but not M1 (33, 43, 59). Together, these studies suggest that CEBPD is a key factor in macrophage polarization, where elevated CEBPD levels induce M1 polarization, and CEBPD inhibition promotes M2 polarization. However, only inflammatory factors and polarization markers were assessed in these studies, while other biological processes that play an important role in macrophage polarization, such as efferocytosis, migration, material and energy metabolism, were not examined. Whether these biological processes are also directly regulated by CEBPD requires further investigation.

### 3.2 Phagocytosis

As a core macrophage function, phagocytosis serves as a defense mechanism to eliminate pathogens, apoptotic cells, and cellular debris to maintain tissue homeostasis and respond to injury (60). Phagocytosis is a complex receptor-mediated process that involves the recognition, engulfment, and degradation of particles larger than 0.5 μm (61). As professional phagocytes, macrophages express two classes of phagocytic receptors that activate signaling pathways resulting in phagocytosis. Opsonic receptors detect host-derived serum proteins bound to target particles. These proteins, known as opsonins, include antibodies, fibronectin, complement, and mannose-binding lectin, which enhance the clearance of particles (61). Non-opsonic receptors, including C-type lectins, lectin-like recognition molecules, and pathogen-associated molecular patterns receptors (such as CD36 and scavenger receptors), directly identify distinct molecular patterns on the particle to be ingested (61). The process of phagocytosis typically involves four phases: (1) detection of the particle, (2) activation of the internalization process, (3) formation of a phagosome, and (4) transformation of the phagosome into phagolysosome (61). For exogenous particles such as pathogens, macrophages typically clear them through non-opsonic receptors-mediated phagocytosis. However, macrophages usually clear endogenous particles such as immune complexes through opsonic receptor-mediated phagocytosis. Low-density lipoprotein (LDL) and liposomes appear to be cleared through opsonic receptor-mediated phagocytosis at low concentrations and non-opsonic receptor-mediated phagocytosis at high concentrations (62, 63).

Recent studies have shown that CEBPD is associated with macrophage phagocytosis. In one study, macrophages with inhibited CEBPD show enhanced phagocytosis (27), indicating that CEBPD is negatively correlated with macrophage phagocytosis. However, another study has shown that CEBPD promotes lipid accumulation in M1 macrophages, but not in M2 macrophages (25). Excessive lipid accumulation in macrophages results from increased uptake of LDL or impaired cholesterol efflux, which causes the formation of lipid-laden foam cells (25). Mechanistically, CEBPD upregulates the prototypic humoral pattern recognition molecule pentraxin 3 (PTX3) expression in M1 macrophages, and PTX3 promotes the opsonic receptor-mediated phagocytosis of LDL by macrophages through binding to C1q or Ficolin-1 (25, 61, 64). In addition, a recent study showed that CEBPD mediates phagocytosis of Aspergillus fumigatus by macrophages through PTX3 (37), but the exact mechanism remains to be further investigated. These data indicate that the CEBPD-PTX3 axis plays an important role in macrophage phagocytosis.

### 3.3 Inflammatory and immune response

CEBPD expression is low under normal physiological conditions and can be rapidly induced by external stimuli (28, 58), suggesting that CEBPD plays an important role in the inflammatory and immune responses to these stimuli. In macrophages, CEBPD directly binds to the promoter and upregulates the expression of inflammatory factors, including C-C motif chemokine ligand 20 (CCL20), CCL3, cyclooxygenase 2 (COX2), C-X-C motif chemokine ligand 1 (CXCL1), interleukin-6 (IL-6), PTX3, S100 calcium-binding protein a8/9 (S100a8/9), and tumor necrosis factor alpha (TNFα) in response to inflammatory stimuli (24, 36, 37, 49, 51). Serum amyloid A 3 (SAA3), an acute-phase protein, is primarily expressed in macrophages and is positively regulated by CEBPD (36). CEBPD directly upregulates the expression of component C3 in macrophages, which plays an important role in the complement system (36). In addition, CEBPD directly upregulates the expression of Toll-like receptors (TLR) 4, 8, and 9 in macrophages to maintain and enhance inflammatory responses (38, 52, 53). On the other hand, CEBPD mediates immunosuppression by directly upregulating the expression of CD274 (also called programmed death ligand 1, PD-L1) and downregulating the expression of the class II major histocompatibility complex transactivator CIITA in macrophages (42, 48). Besides, elevated macrophage CEBPD can directly upregulate the expression of complement C3, coagulation factor X (F10), and serpin family B member 2 (PAI2) to activate the coagulation and complement system, in coordination with inflammation (36). The detailed target genes of CEBPD in macrophages are listed in **Table 2**.

### 3.4 Other biological processes

CEBPD also plays an important role in other biological processes involving macrophages. Macrophage cholesterol efflux substantially protects against the progression of atherosclerosis. In M1 macrophages, increased CEBPD downregulates transmembrane transporter ABCA1 expression, impairing the cholesterol efflux from macrophages and accelerating the development of atherosclerotic lesions (25, 65). Further, CEBPD directly upregulates the expression of NADPH oxidase 1 (NOX-1) and nitric oxide synthase-2 (NOS2) in macrophages to mediate LPS-induced oxidative stress (32, 47). During pyroptosis, activated CEBPD upregulates the expression of gasdermin E (GSDME) and GSDMD to promote macrophage pyroptosis caused by immunoglobulin G (IgG) immune complex treatment (55). In addition, elevated CEBPD binds to the promoter of the osteoclast differentiation marker CTSK gene and upregulates its expression, promoting macrophage differentiation into osteoclasts (28). Recent studies have shown that CEBPD regulates macrophage metabolism. In CEBPD-deficient macrophages, the pyrimidine metabolism pathway is significantly downregulated (31, 66), indicating that CEBPD participates in pyrimidine metabolism. However, the underlying regulatory mechanisms still require clarification.

## 4 Roles of macrophage CEBPD in disease

**Figure 3:**
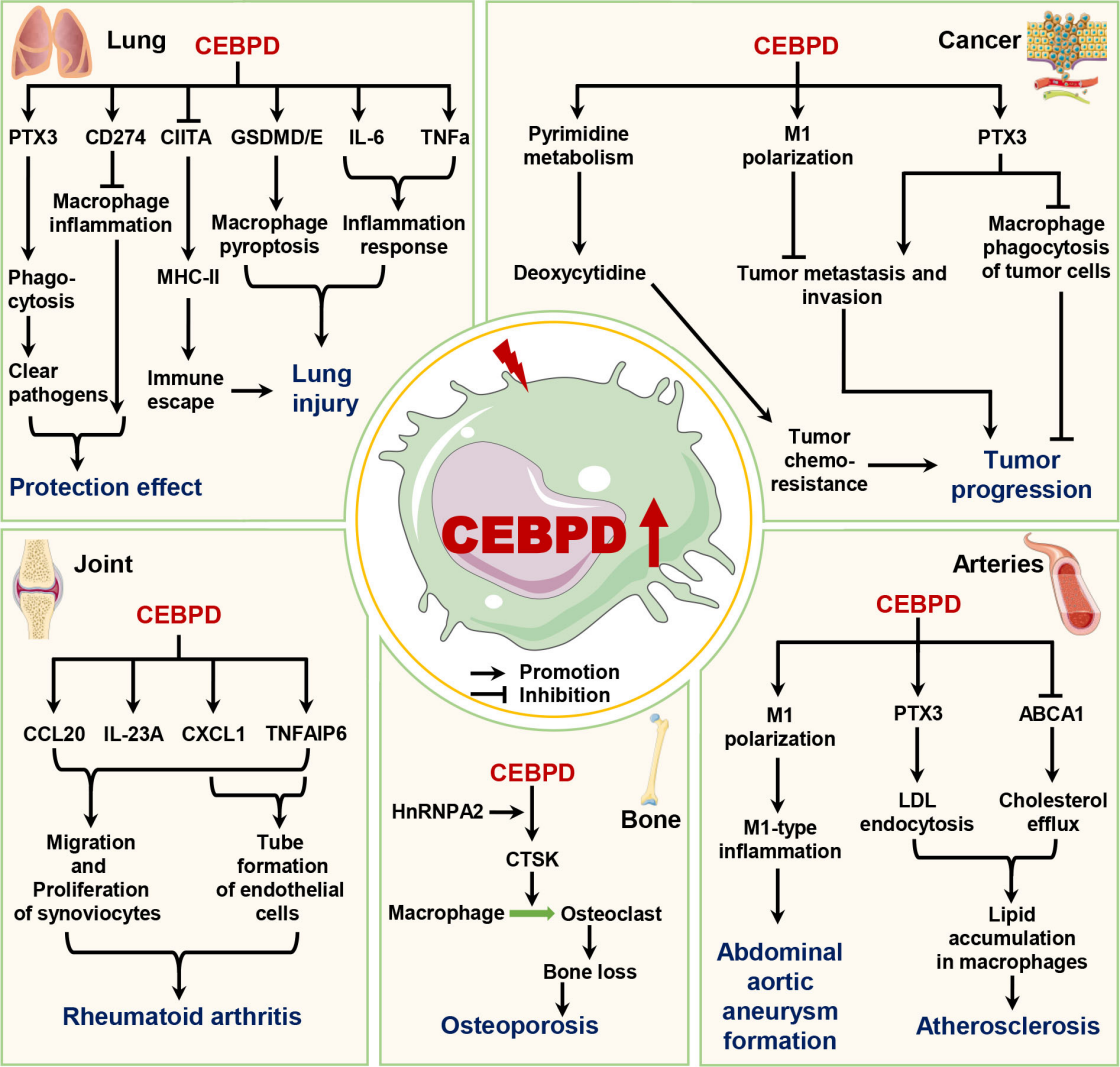
The roles of macrophage CEBPD in diseases. Macrophage CEBPD has different roles in lung diseases and cancers, whereas it can promote the progression of osteoporosis, rheumatoid arthritis, atherosclerosis, and abdominal aortic aneurysm. ABCA1, ATP binding cassette subfamily A member 1; CCL20, C-C motif chemokine ligand 20; CD274, Cluster of differentiation 274; CIITA, Major histocompatibility complex class II trans-activator; CTSK, Cathepsin K; CXCL1, C-X-C motif chemokine ligand 1; GSDMD/E, Gasdermin D/E; HnRNPA2, Heterogeneous nuclear ribonucleoprotein A2; IL-6, Interleukin-6; IL-23A, Interleukin-23A; LDL, Low-density lipoprotein; MHC-II, Major histocompatibility complex class II; PTX3, Pentraxin 3; TNFa, Tumor necrosis factor alpha; TNFAIP6, TNF alpha induced protein 6.

Macrophages participate in tissue homeostasis and disease pathogenesis, whereas macrophage CEBPD is mainly involved in the progression of pathological processes. In this section, we discuss the role of macrophage CEBPD in lung disease, osteoporosis, RA, vascular disease, and tumor biology (**Figure 3**).

### 4.1 Lung diseases

Recent studies have shown that *Klebsiella pneumoniae* (Kp) infection, *Aspergillus fumigatus* infection, and acute lung injury caused by LPS all induce CEBPD expression in lung tissue (37, 54, 67). The bacterial load is increased in the blood of CEBPD-deficient mice compared to wild-type mice after Kp infection, indicating that CEBPD has a role in preventing bacterial spread in mice (67). Further findings indicate that elevated CEBPD caused by both these infections and injury is mainly expressed in macrophages (26, 37, 54, 67). Mechanistically, pathogenic infections induce HuR expression in macrophages to upregulate CEBPD expression, which activates PTX3 by directly binding to its promoter, thereby promoting the phagocytic ability of macrophages toward pathogens (37). Furthermore, in murine lung macrophages with LPS-induced acute respiratory distress syndrome, increased CEBPD upregulates the expression of PD-L1, which inhibits the macrophage-associated inflammatory response to alleviate disease (42).

However, some studies have shown that elevated CEBPD levels in macrophages can exacerbate the progression of lung disease. In acute lung injury caused by LPS, IgG immune complex, or LPS + IgG immune complex, these stimuli increase CEBPD expression via p38/ERK signaling, PI3K/AKT1 signaling, or suppressors of cytokine signaling 3 (SOCS3) pathways (26, 42, 49, 54). High CEBPD levels in macrophages both activate the inflammatory response by directly upregulating the expression of inflammatory factors, such as TNFα, IL-6, and MIP-1/2, and induce macrophage pyroptosis by upregulating the expression of GSDMD/E, which exacerbates the disease process (44, 49, 55). Further, *Mycobacterium tuberculosis* (Mtb) infection or exposure to 19-kDa lipoprotein Mtb components increase CEBPD expression in macrophages (48, 68). Elevated CEBPD levels directly inhibit the CIITA expression that suppresses MHC-II transcription and inhibits macrophage MHC-II Ag presentation, allowing Mtb to evade immune surveillance (48, 69). These studies show that the levels of lung macrophage CEBPD are elevated in response to infection and injury. Elevated macrophage CEBPD facilitates clearance of pathogenic bacteria during acute lung pathogenic infection (e.g., Kp infection). In contrast, in persistent bacterial infections (e.g., Mtb infection), toxins, and immunologic injury, elevated macrophage CEBPD levels exacerbate lung disease progression.

### 4.2 Osteoporosis

A recent study has shown that CEBPD plays vital roles in osteoclastogenesis and bone loss during aging (70). Mitochondrial dysfunction has emerged as an important factor in a wide range of pathologies including aging and osteoporosis. The mitochondria-to-nucleus retrograde signaling (MtRS) pathway originates from dysfunctional mitochondria and causes global nuclear transcriptional reprogramming as its end point, which aids in cellular adaptation to stress (23, 28). Notably, CEBPD is an important factor in the MtRS pathway (23). During aging, hypoxia, or cytochrome c oxidase dysfunction, dysfunctional mitochondria activate the MtRS pathway in macrophages, thereby inducing the expression of heterogeneous ribonucleoprotein A2 (hnRNPA2) and CEBPD (23, 28). HnRNPA2 acts as a co-activator with CEBPD to bind the CTSK promoter, upregulating its expression under hypoxic conditions and promoting macrophage differentiation into osteoclasts (28). However, it should be noted that this enhanced osteoclastogenesis results in bone loss and osteoporosis. In addition, mitochondrial dysfunction caused by cytochrome c oxidase inactivation in macrophages leads to higher levels of cellular and mitochondrial reactive oxygen species, enhanced phagocytosis, boosted inflammatory response, and increased glycolysis (23). This study did not provide evidence of whether these effects were related to elevated CEBPD levels, indicating the need for further research. These studies indicate that elevated macrophage CEBPD promotes the onset and progression of osteoporosis, and targeting macrophage CEBPD is expected to attenuate osteoporosis.

### 4.3 Vascular diseases

CEBPD is known to be involved in the progression of vascular diseases. Lai et al. have reported that CEBPD is mainly expressed in macrophages of atherosclerotic plaques and that CEBPD deficiency in bone marrow cells suppresses atherosclerotic lesions in ApoE^-/-^ mice, indicating that macrophage CEBPD plays a functional role in the pathogenesis of atherosclerosis (25). Further, both M1 and M2 macrophages phagocytose modified LDL; however, CEBPD deficiency reduces the phagocytic ability of M1 macrophages, suggesting that CEBPD is only involved in lipid phagocytosis by M1 macrophages. Further studies have shown that increased CEBPD enhances the lipid accumulation in M1 macrophages through two pathway: (1) directly upregulating PTX3 expression to promote the macropinocytosis of LDL, and (2) downregulating the expression of ABCA1, which impairs the intracellular cholesterol efflux from M1 macrophages (25). In addition, macrophage CEBPD plays a crucial role in the initial inflammatory phase and dominates the key pathogenesis of abdominal aortic aneurysm (AAA) (18). A recent report showed that elevated circular RNA Cdyl promotes AAA formation by inducing M1 macrophage polarization and M1-type inflammation in an angiotensin II (Ang II)- and calcium chloride (CaCl_2_)-induced mouse model of AAA (30). Furthermore, the circular RNA Cdyl acts as a sponge of let-7c to inhibit its expression in macrophages (30). CEBPD is a target of let-7c and plays a vital role in macrophage polarization caused by let-7c (59). Mechanistically, increased CEBPD caused by the upregulated circular RNA Cdyl promotes macrophage M1 polarization and maintains the M1 inflammatory state, which accelerates AAA progression (30). Available research evidence suggests that elevated levels of macrophage CEBPD in vascular tissue can exacerbate the progression of vascular diseases. These studies also suggest that the role of macrophage CEBPD should be considered in studies of other vascular diseases.

### 4.4 Rheumatoid arthritis

CEBPD also plays an important role in RA progression. Chang et al. have reported decreased pannus proliferation and angiogenesis in CEBPD-knockout mice compared with those in WT mice under the collagen-induced RA model (24). In addition, CEBPD is reported to be activated in human RA macrophages (29). Activated CEBPD in macrophages promotes tube formation by endothelial cells and the migration and proliferation of synoviocytes (24). Mechanistically, elevated CEBPD upregulates CXCL1, TNF-alpha-induced protein 6 (TNFAIP6), CCL20, and IL-32A gene transcripts by directly binding to their promoter regions (24). CXCL1 and TNFAIP6 enhance tube formation by endothelial cells, and all four proteins contribute to the migration and proliferation of synoviocytes. Thus, inhibiting macrophage CEBPD expression or activity in the synovial cavity could be a feasible and valuable strategy for RA treatment.

### 4.5 Tumors

Accumulating evidence has shown that macrophage CEBPD plays crucial roles in tumor escape, drug resistance, metastasis, and invasion. Among the cell types present in the tumor microenvironment, tumor-associated macrophages (TAMs) and their precursors account for the largest fraction of the myeloid infiltrates in most solid tumors (71). TAMs produce both tumor-supportive factors and anti-tumor factors that impact tumor growth, angiogenesis, invasion, and metastasis (72). Hsiao et al. have reported that the immunosuppressive prostaglandin E_2_ increases the expression and activity of CEBPD by stimulating the nucleocytoplasmic shuttling of the RNA-binding protein HuR, which binds to and stabilizes CEBPD mRNA in macrophages (27). Then, elevated CEBPD directly upregulates IL-10 and PTX3, which suppresses the ability of macrophages to phagocytose nasopharyngeal carcinoma cells via an autocrine mode of regulation (27). Additionally, antitumor drugs can lead to elevated CEBPD levels in macrophages (31, 50). Increased macrophage CEBPD promotes tumor resistance in two ways: (1) activating the pyrimidine metabolism pathway to produce the low molecular weight free metabolites in macrophages, as in the induction of gemcitabine resistance in pancreatic cancer cells (31); and (2) upregulating PTX3 expression, which enhances the resistance of breast cancer cells to cisplatin and 5-fluorouracil (50). In addition, the macrophage CEPBD/PTX3 axis contributes to metastasis, invasion, and stemness in breast cancer cells (50). Besides, given the important role of macrophage-associated tumor fibrosis in tumor resistance and the association of CEBPD with fibrotic disease (22), additional research is needed to assess whether macrophage CEBPD influences tumor resistance by regulating tumor fibrosis. These studies suggest that macrophage CEPBD plays several important roles in tumor chemoresistance, invasion, metastasis, and stemness maintenance, and can be an effective target for solid tumor therapy.

## 5 Developing therapeutic modulators of CEBPD

As CEBPD plays an important role in a variety of macrophage functions, modifying CEBPD expression or activity may be a promising strategy to treat various diseases. The endogenous CEBPD antagonists ATF3 and FBXW7 were found to inhibit CEBPD expression during monocyte-to-macrophage differentiation at the transcriptional and post-translational levels, respectively, with the inhibitory effect of FBXW7 being stronger (51). Further, CEBPG overexpression in macrophages results in the formation of a heterodimer with CEBPD that inhibits its transcriptional activity (49). However, unlike CEBPD, these proteins are more highly expressed and essential for maintaining normal cellular function, so are not viable therapeutic targets.

### 5.1 Known inhibitors

A growing body of evidence has shown that various compounds inhibit CEBPD expression in macrophages. These compounds are divided into 4 groups: clinical drugs, biological molecules, small-molecule compounds, and natural products (**Table 3**). The antihyperlipidemic drug simvastatin can inhibit CEBPD activity in macrophages to block lipid accumulation, and this inhibitory effect may be mediated by affecting the p38/CEBPD pathway (25). However, the bioavailability and biological half-life of simvastatin are 5% and 3 h, respectively, suggesting a significant rapid pass metabolism in the liver (77). Biological molecules such as carbon monoxide (CO) and miRNA let-7c inhibit CEBPD expression in macrophages treated with LPS (47, 59). Let-7c binds to the 3’-UTR of CEBPD mRNA to inhibit CEBPD expression, but the inhibition mechanism of CO remains unclear. Small-molecule compounds such as the AMPK activator 5-amino-4-imidazole carboxamide riboside (AICAR), MMP2/9 inhibitor inotilone, and HDAC inhibitors SAHA and TSA also inhibit CEBPD expression in macrophages, but the underlying mechanisms require further study (24, 73, 74). Various studies have also shown that treatment with natural products, such as andrographolide, delphinidin, and rosmanol, can inhibit CEBPD expression or activity in macrophages (24, 75, 76).

**Table 3:** Direct and indirect CEBPD inhibitors.

Compound	Effect	References
Clinical drug		
Simvastatin	Simvastatin may inhibit CEBPD activity by p38 pathway in macrophages	(25)
Biological molecules		
Carbon monoxide (CO)	CO inhibits CEBPD expression in macrophages-treated with LPS.	(47)
Let-7c	Let-7c binds to the 3’-UTR of CEBPD mRNA to inhibit CEBPD expression in macrophages	(30, 33, 59)
Small-molecule compounds		
5-amino-4-imidazole carboxamide riboside (AICAR, AMPK activator)	AICAR inhibits nuclear translocation of CEBPD by inhibiting its expression in microglia cells	(73)
Inotilone (MMP2/9 inhibitor)	Inotilone reduces the expression of CEBPD in macrophages	(24)
SAHA (HDAC inhibitor)	SAHA inhibits the mRNA expression of the endogenous CEBPD in macrophages	(74)
TSA (HDAC inhibitor)	TSA inhibits the mRNA expression of the endogenous CEBPD in macrophages	(74)
Natural products		
6-(Methylsulfinyl) hexyl isothiocyanate (6-MITC)	6-MITC inhibits CEBPD expression through suppressing ERK/p38 pathway in macrophages	(46)
Andrographolide	Andrographolide inhibits CEBPD expression in macrophages-treated with LPS	(75)
Arachidin-1	Arachidin-1 inhibits CEBPD expression in macrophages-treated with LPS.	(58)
Delphinidin	Delphinidin may inhibit CEBPD expression through suppressing ERK/p38 pathway in macrophages	(76)
Eicosapentaenoic acid (EPA)	EPA enhances CEBPD expression via site-specific CpG demethylation in its gene promoter in macrophages	(35)
Piceatannol	Piceatannol inhibits CEBPD expression in macrophages-treated with LPS	(58)
Prodelphinidin B2 3,3’ di-O-gallate (PDGG)	PDGG inhibits CEBPD expression in macrophages-treated with LPS	(34)
Resveratrol	Resveratrol inhibits CEBPD expression in macrophages-treated with LPS	(58)
Rosmanol	Rosmanol reduces the expression of CEBPD in macrophages	(24)
Verbenalin	Verbenalin inhibits CEBPD expression in macrophages-treated with IgG IC	(55)

### 5.2 Using molecular modeling to identify putative binding sites

**Figure 4:**
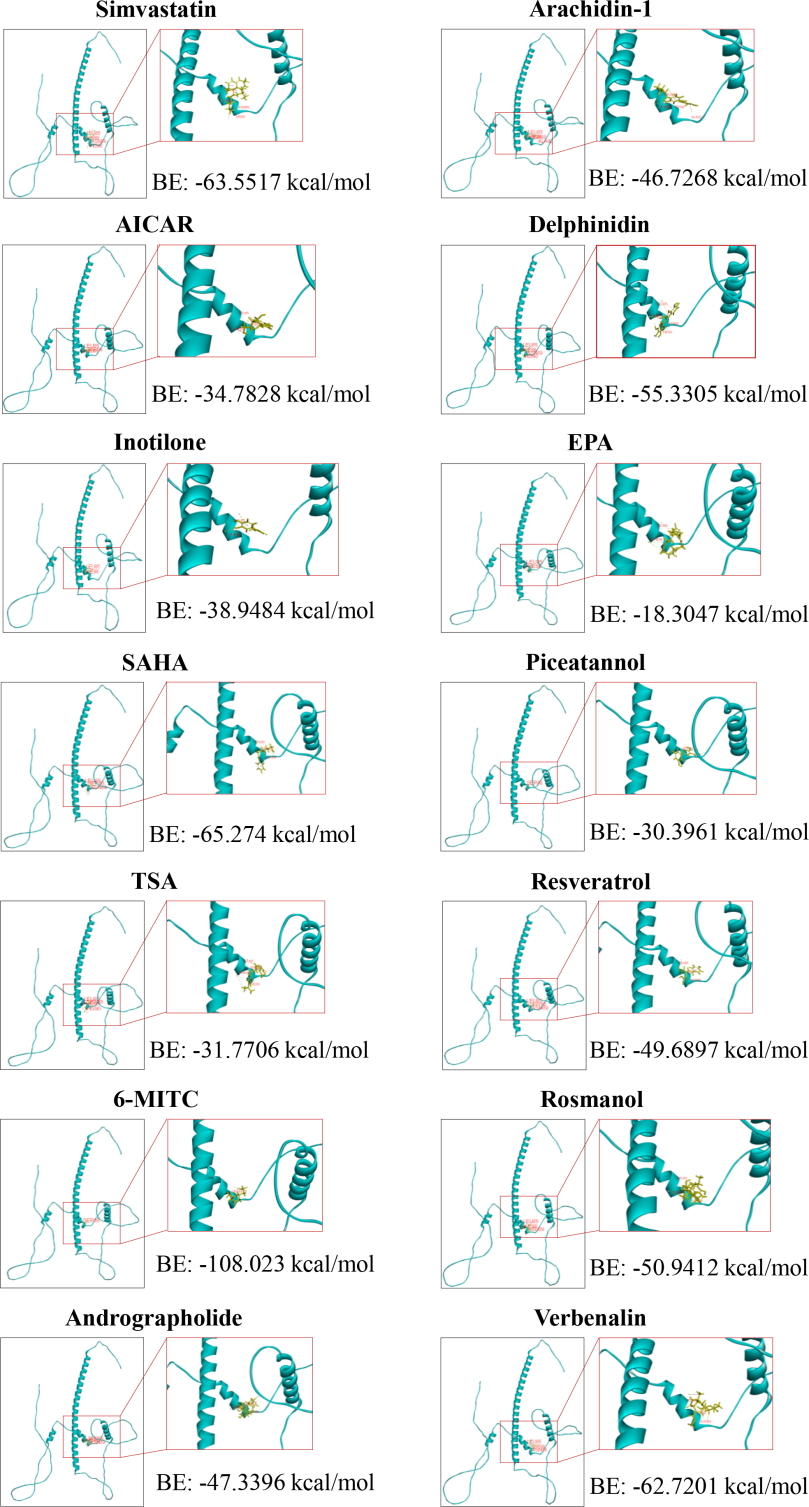
Molecular docking between the transcriptional activation domain of human CEPBD protein and compounds. 6-MITC, 6-(Methylsulfinyl) hexyl Isothiocyanate; AICAR, 5-Aminoimidazole-4-carboxamide-1-β-D-ribofuranoside; EPA, Eicosapentaenoic acid; SAHA, Suberoylanilide hydroxamic acid; TSA, Trichostatin A.

We have analyzed the ability of these inhibitors to bind to the CEBPD protein using molecular docking software (Discovery Studio). All compounds, except prodelphinidin B2 3,3’ di-O-gallate (PDGG) and CO, are predicted to bind to CEBPD protein (human), with binding energies of less than -18 kcal/mol (**Figure 4**, **Supplementary Figure S1**). Binding energy < 0 indicates that a compound can spontaneously bind to the target protein, and increasingly negative free binding energies result in the formation of stronger complexes between the compound and the target protein (78). Further, we predicted that these compounds bind to the helix 2 (H2) in N-terminal transcriptional activation domain (TAD) and H3 in middle regulatory domain (RD) of CEBPD (**Figure 1B**). The 11 main points of contact of CEBPD are Leu 85, Phe 86, Ser 88, Asn 89, Pro 123, Asp 124, Trp 125, Gly 126, Ala 130, Leu 134, and Gln 138 (**Figure 4**, **Supplementary Figure S1**). We comparatively analyzed the protein sequences of human CEBP family members with Clustal Omega (**Figure 1B**). All members of the CEBP family possess a variable N-terminal region and a highly conserved C-terminal (>90% sequence identity) containing a bZIP domain (22). The inhibitors we evaluated are predicted to bind to target the TAD and RD domain, not the bZIP domain of CEBPD (**Figure 4**, **Supplementary Figure S1**), suggesting that these compounds are less likely to bind other CEBP family members. Moreover, among CEBP family members, only four of the 11 critical binding residues predicted for CEBPD (Leu 85, Phe 86, Pro 123, and Gln 138) are also present only on CEBPA (**Figure 1B**). Among these compounds, only eicosapentaenoic acid (EPA) binds both Leu 85 and Phe 86 when it binds H2 in the TAD domain (**Figures 1B**, **4**). These results suggest that with the exception of EPA which may have some effect on CEBPA activity, the remaining compounds have no effect on other members of the CEBP family. Further, we found that these 14 compounds do not bind to CEBPG and CEBPZ. In these 14 compounds, only simvastatin, 6-(methylsulfinyl) hexyl isothiocyanate (6-MITC), and verbenalin have only one binding site with CEBPA, CEBP, and CEBPE and their binding energies with these members are all lower than that with CEBPD (**Supplementary Figure S2**). The above results indicate that these compounds are predicted to inhibition of primarily affect CEBPD activity in macrophages. The exact mechanisms of these compounds require further study.

### 5.3 Towards macrophage-specific delivery of inhibitors

When used, these drugs often inhibit CEBPD and other proteins in other types of cells, causing side effects, indicating the importance of designing macrophage-specific delivery systems. One such delivery system is clodronate liposomes, which are used to deplete macrophages in a temporally controlled manner in many research laboratories worldwide, suggesting that liposomes may be ideal vehicles for macrophage phagocytosis (79). Liposomes are spherical vesicles comprising one or more concentric phospholipid bilayers enclosing an aqueous core, and have been used as nontoxic and biodegradable delivery systems for several drugs, such as amphotericin B, doxorubicin, and siRNA, to treat fungal infections, Kaposi’s sarcoma, and ovarian cancer, respectively (80, 81). Mechanistically, liposomes are recognized as foreign particles by phagocytes, especially macrophages, and are ingested as an internal vesicle called phagosome. The ingestion is followed by a fusion of the phagosome with lysosomes containing phospholipases, which disrupt the phospholipid bilayer of liposomes, releasing its contents inside the cells/macrophages (82). Therefore, biological molecules or small-molecule compounds such as let-7c or andrographolide can be encapsulated into liposome vesicles. After liposomes are phagocytosed by macrophages, these molecules or compounds could regulate CEBPD expression or activity, thereby modulating macrophage function. However, accelerated blood clearance and non-specific targeting are the main drawbacks of the liposome delivery system. Despite years of in-depth research in these areas, progress has been slow.

Alternatively, the specificity of other drug delivery systems, such as lipid nanoparticles (LNP), has shown promising applications in targeting macrophage regulation (83, 84). LNPs are a class of lipid vesicles that have gained recognition as highly effective carriers for delivering diverse therapeutic agents due to their biocompatibility, loading capacity, and customizable features (85). LNPs consist of four main lipid components with essential functions: ionizable lipids, helper lipids, cholesterol, and polyethylene glycol (PEG) lipids (85). In recent years, LNPs have emerged as a highly promising delivery platform for gene-based therapies in various diseases, such as cardiovascular disease (85, 86). Recently, Du et al. formulated 1,2-dioleoyl-sn-glycero-3-phospho-l-serine-doping ALC-0315 (DOPS, an ionizable lipid) LNP that successfully generated chimeric antigen receptor-macrophages capable of selectively engaging and phagocytosing activated cardiac fibroblasts in ischemia-reperfusion mouse hearts (86). To target macrophages, DOPS is recognized by scavenger receptors highly expressed on the surface of macrophages (87). These studies suggest that DOPS-LNP containing CEBPD inhibitors is an effective potential delivery vehicle for regulating macrophage function.

## 6 Summary and perspectives

Recent technological advances in high-throughput sequencing have allowed us to understand macrophage heterogeneity across development, health, and different disease conditions with unprecedented depth and perspective, highlighting the irreplaceable role of macrophages in physiology and disease progression. However, there are no effective strategies for targeting macrophages to treat diseases. Despite the identification of many distinct cellular states of macrophages, we are only beginning to understand whether these states represent functionally distinct macrophage subsets determined by their ontogeny or whether plasticity allows them to switch between functional states (61). In this review, we explored the feasibility of targeting macrophages for disease treatment, using CEBPD as an example. CEBPD expression is low in macrophages under physiological conditions, but it can be rapidly induced by external stimuli (35, 37, 42, 46). We have summarized a broad range of recent advances in research on macrophage CEBPD and its roles in various diseases. These studies suggest that modulating macrophage function by targeting CEBPD is an attractive strategy for disease treatment.

Despite the encouraging progress in exploring the relationship between CEBPD and macrophages, some critical questions must be answered before targeting macrophage CEBPD for clinical use in disease treatment. Macrophages used in the aforementioned studies were derived from the mouse macrophage cell line RAW264.7, the human monocytic leukemia cell line THP-1, the human peripheral blood mononuclear cells, or the mouse bone marrow. These macrophages are either immortalized or induced rather than isolated from tissues. Although *in vitro* methods are easy to evaluate and readily provide data related to biological mechanisms, they lack the ability to *in vivo* biokinetics and cross-talk between tissues, potentially leading to a misinterpretation of *in vitro* data (88). It is possible that in *in vivo* systems, the cells are exposed to lower concentrations, since chemical compounds cannot directly reach the target cells. On the other hand, drugs may accumulate in certain organs, tissues, or cells, resulting in a prolonged or enhanced exposure of the target tissue. Another consideration is that macrophages respond differently to drugs or stimuli under the influence of other cells in the tissue microenvironment. Further studies should focus on testing and validating the role of macrophage CEBPD in the tissues of patients and/or model animals. Additionally, although evidence shows that CEBPD levels are elevated in macrophages under *in vitro* stimuli, the expression patterns of CEBPD in distinct macrophage subsets during physiological states and different disease states remain unclear. Furthermore, Tanaka et al. have reported that 35% of CEBPB^-/-^ mice and 85% of CEBPB^-/-^; CEBPD^-/-^ mice die soon after birth, suggesting that CEBPD and CEBPB have overlapping functions to some degree (89). However, Spek et al. have shown that the expression of other CEBP family members, except for an increase of CEBPA, shows no changes in CEBPD knockout RAW264.7 cells under LPS stimulation, compared to that in wild type cells (66). We treated bone marrow-derived macrophages from macrophage-specific knockout CEBPD mice with LPS and the results were similar to those of the aforementioned study (Unpublished). Further investigations are thus required to determine whether CEBPA and CEBPD are functionally complementary in macrophages. The evidence summarized in this review strengthens the hypothesis that CEBPD may be an effective target for modulating macrophage function to treat disease and serves as a reference for further investigation in this field.
