# Supplementary material for: The feasibility of targeting macrophage for disease treatment: roles of CEBPD
Source: Front Immunol. 2025 Sep 8;16:1650161. doi: 10.3389/fimmu.2025.1650161 (PMC12450702; doi:10.3389/fimmu.2025.1650161)
Supplement: Supplementary file 1 [file Table1.docx]

**Supplementary Figure legends:**

**Supplementary Figure 1. Molecular docking between the regulatory domain (RD) of human CEPBD protein and compounds.** 6-MITC: 6-(Methylsulfinyl) hexyl Isothiocyanate; AICAR: 5-Aminoimidazole-4-carboxamide-1-β-D-ribofuranoside; EPA: Eicosapentaenoic acid; SAHA: Suberoylanilide hydroxamic acid; TSA: Trichostatin A

**Supplementary Figure 2. Molecular docking between the other human CEPB family members besides CEBPD and three compounds.**

**Supplementary Table 1. Experimental conditions that regulation of CEBPD activity to show discrepancies**

| **Experimental conditions** | **CEBPD Changes** | **Effects** | **References** |
| --- | --- | --- | --- |
| **In vivo experiments with CEBPD knockout mice** | | | |
| Collagen-induced arthritis | CEBPD deficiency | CEBPD knockout Reduced pannus formation and greater integrity of joint architecture in affected paws | 24 |
| ApoE^-/-^ Atherosclerosis model | CEBPD deficiency | CEBPD gene deficiency in bone marrow cells suppressed atherosclerotic lesions | 25 |
| LPS induced acute lung injury | CEBPD deficiency | Serum and BALF S100A8/A9 levels were decreased and BALF neutrophil recruitment as well as IL-1α and IL-6 levels were significantly decreased in CEBPD knockout mice | 51 |
| **In vitro experiments-bone marrow-derived macrophages (BMDMs)** | | | |
| Single-cell multi-omics profiling on bone marrow samples from mice of different ages (1, 6, and 20 months) | CEBPD increased | The mRNA level of CEBPD was increased in bone marrow macrophages with increased osteoclast differentiation during the aging process | 70 |
| Treatment BMDMs with TNF-α | The CEBPD level was increased | Upregulates CCL20, CXCL1, IL23A, TNFAIP6 expression | 24 |
| Treatment M1-BMDMs from CEBPD knockout mice with oxLDL or acLDL | The CEBPD level was increased | Inhibits PTX3 expression and increases ABCA1 expression | 25 |
| Treatment M1-BMDMs with exosomes derived from adipose-derived mesenchymal stem cell | The CEBPD level was inhibited | Inhibited CEBPD suppressed M1 polarization | 33 |
| M1-BMDMs knocked out CEBPD by siRNA | The CEBPD level was inhibited | Knockdown of CEBPD enhanced the expression of M2 phenotype | 59 |
| Treatment BMDMs with LPS | The CEBPD level was increased | Increased CEBPD expression to enhance the expression of Nox-1 | 32 |
| Treatment BMDMs from WT or CEBPD knockout mice with LPS | CEBPD deficiency | CEBPD gene deficiency inhibited the expression of IL-6 | 36 |
| Treatment BMDMs from WT or CEBPD knockout mice with LPS | CEBPD deficiency | CEBPD gene deficiency enhanced the expression of TNF-α and MCP-1 | 66 |
| **In vitro experiments-RAW264.7 cells** | | | |
| RAW264.7 cells were treated with LPS | The CEBPD level was increased | Increased CEBPD enhanced the PD-L1 expression | 42 |
| Treatment RAW264.7 cells with LPS | The CEBPD level was increased | Increased CEBPD inhibited the expression of FBXW7α | 38 |
| RNA-Seq analysis of CEBPD-deficient RAW264.7 cells | CEBPD deficiency | The genes of pyrimidine metabolism pathway were downregulated in CEBPD-deficient macrophages | 31 |
| RAW264.7 cells knockdown CEBPD by siRNA then were treated with IgG immune complex | The CEBPD level was inhibited | The expressions of TNF-α, MIP-2 and 1α were downregulated | 44 |
| Knockdown of subunit IVi1 of Cytochrome c oxidase in RAW264.7 cells with shRNA | Enhanced the nuclear level of CEBPD | Increased inflammatory cytokines and the phagocytic activity of macrophages | 23 |
| Treatment RAW264.7 cells with hypoxic conditions | Enhanced the transcriptional activity of CEBPD | Increased the expression of cathepsin K to induce osteoclast differentiation | 28 |
| Treatment RAW264.7 cells transfected with let-7c mimic with LPS +INF-γ | The CEBPD level was inhibited | Let-7c mimic inhibits CEBPD gene expression to suppress M1 polarization | 30 |
| M1 RAW264.7 cells were transfected with CEBPG overexpression plasmid | The CEBPD level was inhibited | Inhibited CEBPD suppressed the expression of TNF-α and IL-6 | 49 |
| RAW264.7 cells were transfected with SOCS3 overexpression plasmid, then were treated with IgG immune complex | The CEBPD level was increased | Increased CEBPD enhanced the generation of pro-inflammatory mediators | 26 |
| Treatment RAW264.7 cells with Mycobacterium tuberculosis 19-kDa lipoprotein | The CEBPD level was increased | Increased CEBPD enhanced the expression CIITA | 48 |
| Treatment LPS induced RAW264.7 cells with 6-MITC | The CEBPD level was inhibited | Inhibited CEBPD suppressed the expression of COX-2 | 46 |
| Treatment LPS induced RAW264.7 cells with delphinidin | The CEBPD level was inhibited | Inhibited CEBPD suppressed the expression of COX-2 | 75 |
| Treatment LPS induced RAW264.7 cells with PDGG | The CEBPD level was inhibited | Inhibited CEBPD suppressed the expression of COX-2 | 34 |
| Treatment serum-stimulated RAW264.7 cells with bovine type I collagen | The CEBPD level was increased | Increased CEBPD enhanced the expression of COX-2 | 39 |
| Treatment LPS induced RAW264.7 cells with andrographolide | The CEBPD level was inhibited | Inhibited CEBPD suppressed the inflammation | 74 |
| Treatment LPS induced RAW264.7 cells with arachidin-1, piceatannol, resveratrol | The CEBPD level was inhibited | Inhibited CEBPD suppressed the inflammation | 58 |
| Treatment RAW264.7 cells with LPS or/and carbon monoxide (CO) | The CEBPD level was inhibited | CO inhibited COX-2 and NOS-2 expression induced by LPS through suppressing CEBPD | 47 |
| RAW264.7 cells overexpressed let-7-5p were treated with LPS and INF-γ | The CEBPD level was inhibited | Inhibited CEBPD suppressed M1 polarization and promote M2 polarization | 43 |
| **In vitro experiments-THP-1 derived macrophages** | | | |
| Knocked down or overexpressed CEBPD in THP-1-induced macrophages treated with A. fumigatus conidia | The CEBPD level was increased or inhibited | The expression of PTX3 inhibited and increased to modulate macrophage phagocytosis | 37 |
| Treatment THP-1- derived macrophages with PGE_2_ | The CEBPD level was increased | Increased CEBPD enhanced the expression IL-10 and PTX3 | 27 |
| Treatment THP-1- derived macrophages with R848 | The CEBPD level was increased | Increased CEBPD enhanced the expression of TLR8 | 52 |
| Treatment THP-1- derived macrophages with LPS | The CEBPD level was increased | Increased CEBPD enhanced the expression of ALOX5AP | 45 |
| **In vitro experiments-** **MH-S cells** | | | |
| CEBPD in mouse alveolar macrophage line MH-S cells knocked out by siRNA, then were treated with LPS | The CEBPD level was inhibited | Inhibited the expression of TNF-α and IL-6 induced by LPS | 54 |
| CEBPD in mouse alveolar macrophage line MH-S cells knocked out by siRNA, then were treated with IgG immune complex | The CEBPD level was inhibited | Inhibited CEBPD suppressed the expression of GSDME and GSDMD | 55 |
| **In vitro experiments-** **other types of macrophages** | | | |
| CEBPD knockout Hoxb8 cells treated with LPS +INF-γ | CEBPD deficiency | The expressions of S100A8/A9 were inhibited | 51 |
| Treatment LPS induced primary astrocytes and BV2 cells with AICAR | The CEBPD level was inhibited | Inhibited CEBPD suppressed the inflammation | 73 |
