# Supplementary figures and images for: The feasibility of targeting macrophage for disease treatment: roles of CEBPD
Source: Front Immunol. 2025 Sep 8;16:1650161. doi: 10.3389/fimmu.2025.1650161 (PMC12450702; doi:10.3389/fimmu.2025.1650161)

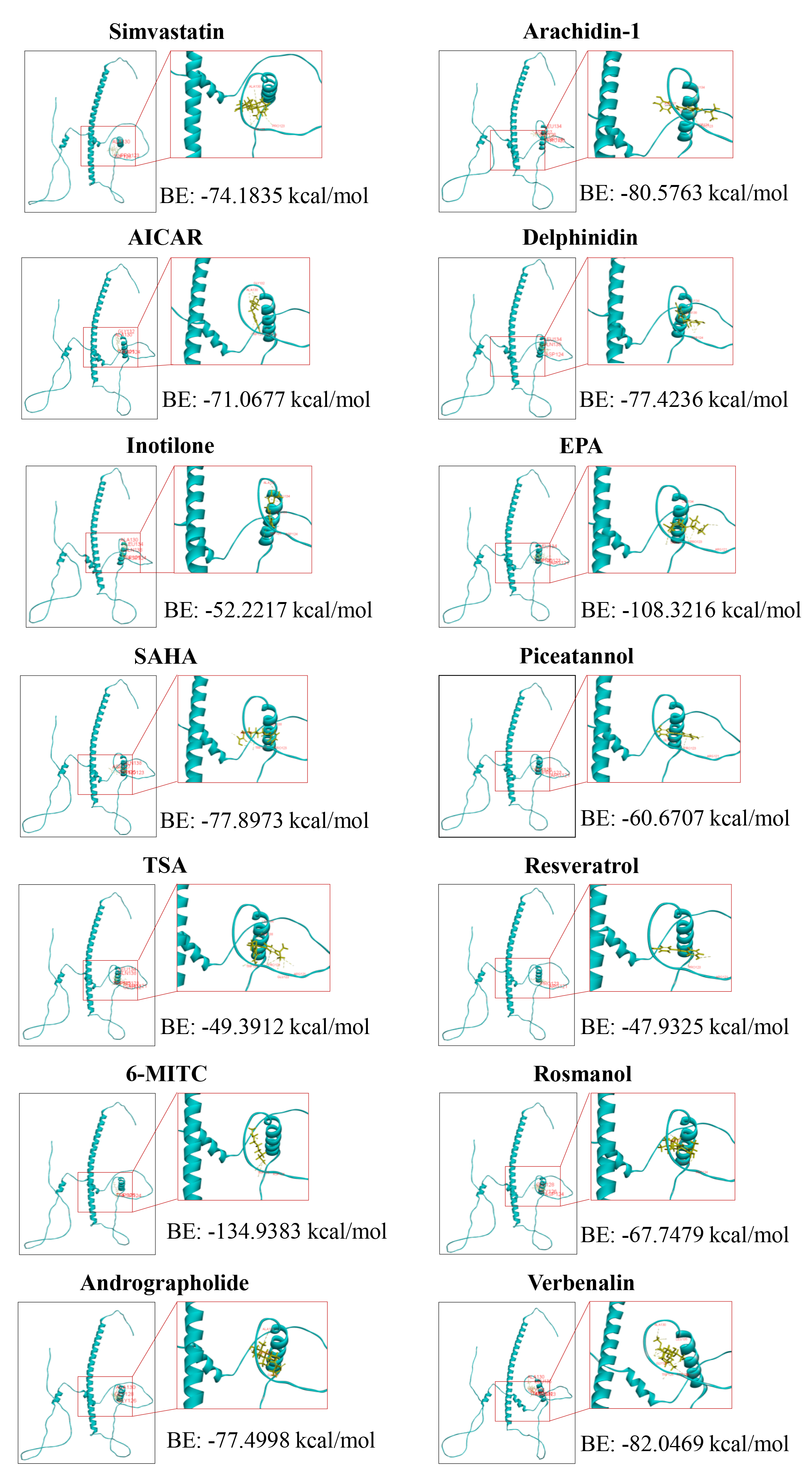

Supplement: Supplementary file 2 [file Image1.tif]

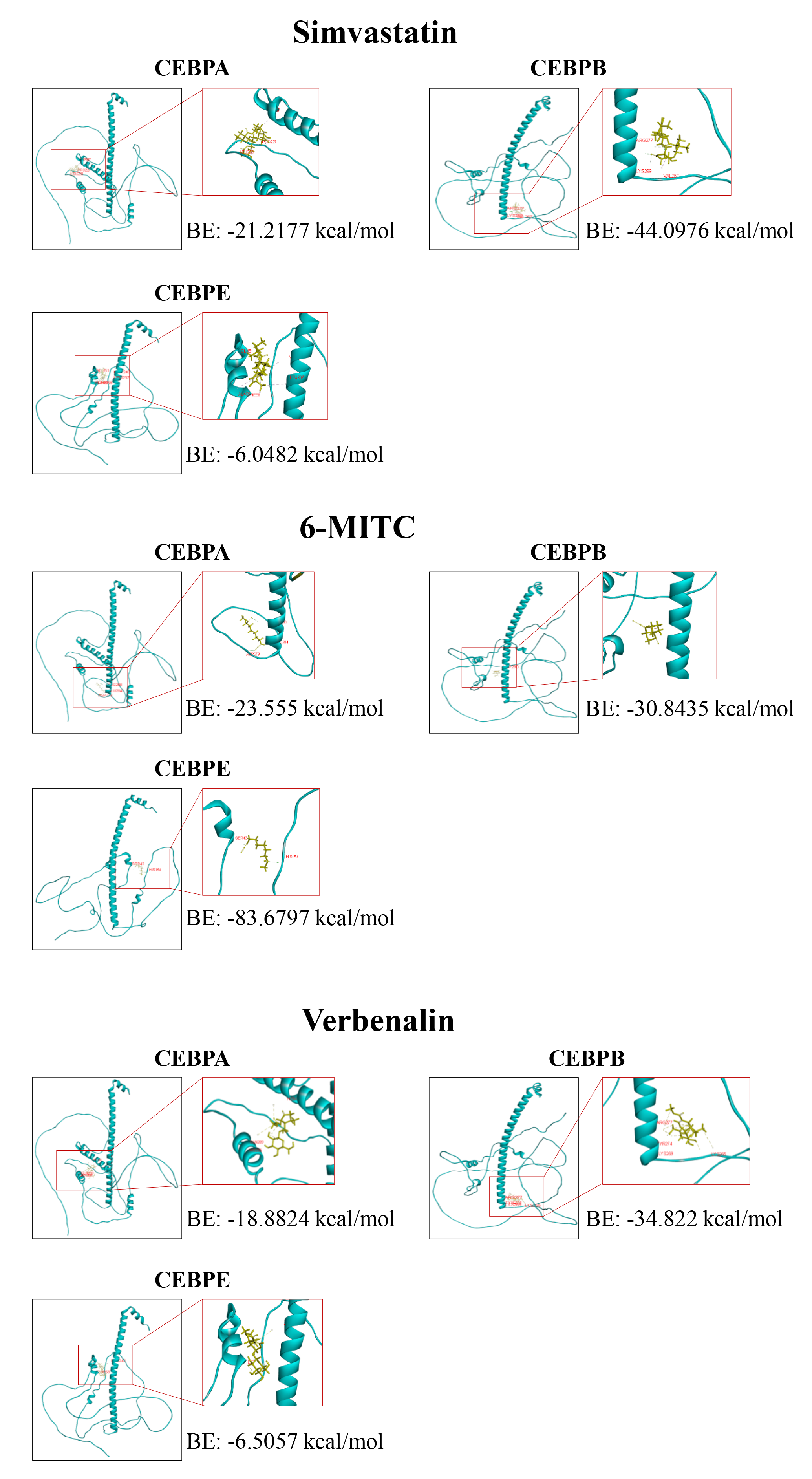

Supplement: Supplementary file 3 [file Image2.tif]
